# Editorial: Reviews in psychiatry 2022: child and adolescent autism

**DOI:** 10.3389/fpsyt.2023.1317073

**Published:** 2023-11-07

**Authors:** Wael Askri, Asma Bouden

**Affiliations:** ^1^Faculty of Medicine, Tunis El Manar University, Tunis, Tunisia; ^2^Department of Child and Adolescent Psychiatry, Razi Hospital, Mannouba, Tunisia

**Keywords:** autism spectrum disorder, child and adolescent, systematic review, meta-analysis, novelties

Autism Spectrum Disorder (ASD) is a complex neurodevelopmental disorder characterized by difficulties in social communication, repetitive behaviors, and sensory issues.

The objective of this Research Topic was to highlight recent advances in the field. Eight published papers delving into original issues were collected. These issues included the experience of college students with ASD, the impact of parental resolution to diagnosis; innovative interventions such as mindfulness, non-invasive neurostimulation and physical-exercise based programs; the intricacies of visual information processing; and the association between labor epidural analgesia and the risk of ASD.

## Transitioning to college: the autistic experience

Austermann et al. Conducted a study involving 40 autistic college students referred as twice exceptional students with ASD (2easd), and examined their experience transitioning from high school to college. Semi-structured interviews were interested in the postsecondary transition process, identifying the factors that these students regarded as best practices, and the challenges they encountered when transitioning to their postsecondary institutions. They reported active participation in selecting their colleges, but their high school experience left much to be desired in terms of robust transition services. Participants expressed a strong desire for more opportunities in high school to develop executive-function skills and enhance their social interactions. Moreover, they emphasized the importance of gaining greater independence during their high school years.

## Parental resolution to diagnosis

Naicker et al. conducted a systematic review on 592 parents. They included 14 papers that investigated parental resolution or acceptance of an ASD diagnosis. Authors identified six factors that influence parental resolution and acceptance of autism diagnosis. Negative emotions such as grief, denial, shame and blame negatively impacted the resolution process. Severity of symptoms; religious and cultural beliefs; knowledge; positive emotions; and social support were associated with greater resolution. Most of the studies were qualitative and only one randomized control trial was included.

## Non-invasive neurostimulation and ASD

Zhang and Zhang systematically reviewed 22 studies with 552 patients enrolled. Transcranial magnetic stimulation (TMS) or transcranial direct current stimulation (tDCS) were used. Many studies reported a positive effect of NINS in management of ASD social, cognitive and behavioral symptoms. However, authors reported a very important heterogeneity and variability between studies in terms of patient's profiles, study designs, stimulation protocols and outcome measurements. Ten studies showed low bias risk, while seven had a high bias risk, and five had a moderate bias risk.

## Exercise interventions for children with ASD

Toscano et al. Aimed to examine the effects of physical activity on symptoms and associated comorbidities in ASD. They conducted a non-randomized controlled trial including 229 patients. Authors highlighted that the structured exercise program considerably diminished social interaction problems, attention deficit, emotional reactivity, verbal and motor stereotypies and sleep disturbances. However, physical exercise did not affect eye contact and food selectivity. Despite methodological limitations, authors recommended that structured, personalized exercise programs should be a complementary therapy in ASD.

The delay in fundamental motor skills (FMS) is a significant concern among children with ASD. Ji et al. conducted a systematic review and meta-analysis in order to evaluate the effectiveness of exercise interventions in improving FMS in this population. Thirteen randomized control trials of exercise interventions were included, of which 10 underwent meta-analysis. In these studies, the outcome measures were assessed using validated tools including: the Bruininks-Oseretsky Test of Motor Proficiency-2, Test of Gross Motor Development-2 and the movement assessment battery for children-2. These tools evaluated locomotor skills (LMS), object control skills (OCS), and stability skills (SS). Authors demonstrated the effectiveness of exercise interventions in improving FMS, with moderate to large effect sizes observed in LMS, OCS, and SS. This study found also a significant difference between used measurement tools.

## Understanding visual information processing

Children with ASD manifest abnormalities in visual orientation, continuous visual exploration, and visual-spatial perception. Zhou et al. hypothesized that abnormal visual perception in ASD is related to the abnormal visual information transmission and abnormal development of visual cortex. In their study, they reviewed the main manifestations and underlying mechanisms of abnormal visual perception in ASD, including abnormal transmission of P100, and abnormal visual information transmission in the retina-lateral geniculate nucleus-visual cortex pathway.

## Mindfulness-based interventions

Loftus et al. systematically reviewed 23 articles in order to examine the efficacy of mindfulness-based interventions for improving psychological wellbeing, anxiety, social skills, and aggressive behaviors in children and young people (CYP) with ASD. These studies used movement or mind-based mindfulness interventions. Twelve studies modified the intervention protocols in order to suit specific needs of CYP with ASD. Positive effects of mindfulness-based interventions on CYP with ASD was reported in the majority of findings. Authors reported that these interventions could be implemented by trained parents and educators with the same efficacity compared to clinicians and therapists. Out of 23 studies, four were strong, five were adequate, and 14 were weak methodologically. Thus, authors recommended further rigorous research.

## Labor epidural analgesia (LEA) and ASD risk

Wang et al. conducted a systematic review and meta-analysis based on cohort study in order to evaluate the association between LEA exposure during parturition and ASD in newborns. Five studies were included with 1,763,454 participants in the USA. The studies included were retrospective cohorts. All of them were of high quality based on the assessment using the Newcastle-Ottawa Scale. All the studies found an association between LEA exposure and ASD. In the meta-analysis, authors reported a statistically significant correlation between LEA and ASD, even after eliminating the study source of heterogeneity, suggesting the stability and reliability of the findings. Also, after adjusting for potential confounders, the results still highlight that LEA exposure was associated with increased ASD risk in children. The findings were limited by the limited number of studies included, the lack of diversity of the study population, and the heterogeneity in results. However, authors suggest that newborns LEA exposed should be screened as early as possible for ASD.

## Conclusion

This Research Topic helps highlighting recent advances in the field of ASD, providing valuable insights into the experiences of autistic individuals and their families, effective interventions, sensory abnormalities, and risk factors such as LEA exposure. These areas may represent interesting frameworks to develop research approaches.

## Author contributions

WA: Writing—original draft. AB: Supervision, Writing—original draft.

